# A DNA Mini-Barcoding System for Authentication of Processed Fish Products

**DOI:** 10.1038/srep15894

**Published:** 2015-10-30

**Authors:** Shadi Shokralla, Rosalee S. Hellberg, Sara M. Handy, Ian King, Mehrdad Hajibabaei

**Affiliations:** 1 Biodiversity Institute of Ontario and Department of Integrative Biology, University of Guelph, Guelph, ON N1G 2W1, Canada; 2 Department of Microbiology, Mansoura University, Mansoura 35516, Egypt; 3 Chapman University, Schmid College of Science and Technology, Orange, CA 92866, USA; 4 Office of Regulatory Science, Center for Food Safety and Applied Nutrition, U.S. Food and Drug Administration, College Park, MD 20740, USA

## Abstract

Species substitution is a form of seafood fraud for the purpose of economic gain. DNA barcoding utilizes species-specific DNA sequence information for specimen identification. Previous work has established the usability of short DNA sequences—mini-barcodes—for identification of specimens harboring degraded DNA. This study aims at establishing a DNA mini-barcoding system for all fish species commonly used in processed fish products in North America. Six mini-barcode primer pairs targeting short (127–314 bp) fragments of the cytochrome c oxidase I (*CO1*) DNA barcode region were developed by examining over 8,000 DNA barcodes from species in the U.S. Food and Drug Administration (FDA) Seafood List. The mini-barcode primer pairs were then tested against 44 processed fish products representing a range of species and product types. Of the 44 products, 41 (93.2%) could be identified at the species or genus level. The greatest mini-barcoding success rate found with an individual primer pair was 88.6% compared to 20.5% success rate achieved by the full-length DNA barcode primers. Overall, this study presents a mini-barcoding system that can be used to identify a wide range of fish species in commercial products and may be utilized in high throughput DNA sequencing for authentication of heavily processed fish products.

Food fraud from species substitution is an emerging risk given the increasingly global food supply chain and potential food safety issues. Economic food fraud is committed when food is deliberately placed on the market, for financial gain, with the intention of deceiving the consumer^1^. As a result of increased demand and the globalization of the seafood supply, more fish species are being encountered in the market^2^. In fact, the Seafood List from the U.S. Food and Drug Administration (FDA) contains more than 1,700 acceptable market names that can be used to label seafood in interstate commerce in the U.S.^3^. Subsequently, the need for accurately labelled food products and full disclosure of product composition has become more critical^4^,^5^. One difficulty in this is the authentication process of different seafood products through examination of the physical appearance of specimens. In their whole or unprocessed form, these species can generally be identified based on morphological indicators; however, over half of the fresh/frozen finfish imported into North America is processed from its original form into products such as fillets and steaks, blocks, and fish sticks^3^. Moreover, species identification by morphological indicators requires a certain level of expertise to distinguish between closely related species. Unfortunately, most consumers are unable to detect cases of mislabelling or fraud given that recognizable external morphological features are typically removed when the fish is processed^4^.

To audit and prevent species fraud on the commercial market, a number of molecular methods have been developed, including use of a unique protein or DNA profiles found in different species^6^. DNA barcoding provides a rapid, cost-effective method for accurate identification at the species-level through comparative analysis of sequence variation in a short, standardized fragment of the genome^7^. The designated DNA barcode for animal species identification is a ~650-bp fragment of the mitochondrial gene coding for cytochrome c oxidase 1 (*COI*)^5^,^8^. A number of studies have shown the applicability of DNA barcoding for accurate identification of a wide range of fish species^9^,^10^. Recently, DNA barcoding has been employed as a species identification tool for food authentication and safety concerns, including incorrect product labelling^11^,^12^, ingredient substitutions^2^ or food contamination^6^,^13^, as well as for regulatory use^14^. DNA-based methods can also be used to monitor illegal trading involving protected or endangered species^5^,^15^,^16^ or to identify the species origin of commercially processed food^13^,^17^,^18^. However, some of the processing and preservation methods used with seafood products are not conducive to DNA barcoding with the full-length target gene region^19^,^20^,^21^. DNA degradation has been recognized as a considerable limitation in DNA-based analyses of these samples, and PCR amplification of full-length (i.e., ~ 650 bp) barcodes from moderately or highly processed samples is significantly challenging. In addition, processed seafood products often contain multiple additives, preservatives, and flavors that may affect the quantity and quality of DNA extracted from these products^21^,^22^,^23^. Alternatively, a mini-barcoding approach, which focuses the analysis on shorter DNA fragments (e.g., 100–200 bp) within the full-length barcode, has been shown to be effective in obtaining DNA sequence information from specimens containing degraded DNA^24^,^25^. The sequencing information generated from a small (≥100 bp) mini-barcode fragment of *COI* within the full-length DNA barcode region can provide the information required for identification of individual species with more than 90% species resolution^21^,^24^,^26^. However, extensive mini-barcode primer development and testing specifically for use with commercially processed fish species has not been carried out.

Here, we designed and optimized multiple primer sets to amplify mini-barcodes within the *COI* barcode region. These mini-barcode primer sets were then used to identify species in a variety of commercially processed fish products obtained in the United States.

## Materials and Methods

### Sample collection

**Figure 1:**
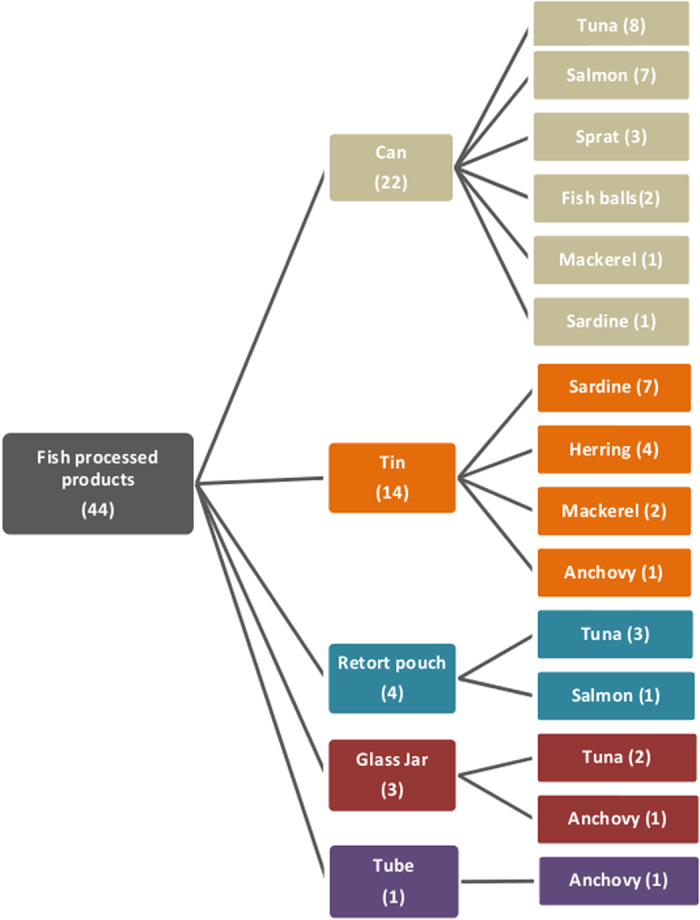
Commercial fish products used for DNA mini-barcoding authentication.

A total of 96 authenticated fish muscle tissue samples were obtained, representing 88 different species. The fish tissue samples were supplied by the FDA-Center for Food Safety and Applied Nutrition (Supplementary Materials-Table S1). These samples are from the FDA’s Reference Standard Sequence Library for Seafood Identification (http://www.fda.gov/Food/FoodScienceResearch/DNASeafoodIdentification/ucm238880.htm) and all are linked to authenticated, vouchered specimens. These samples were used for construction of a DNA barcode library, as described below. Also they were used for optimization of mini-barcode primers designed in this study. For analysis of mini-barcode primers with commercial products, a total of 44 heavily processed seafood products representing a variety of species and product types were purchased in the United States in May 2012 from online retail sources (Fig. 1). Subsamples were collected from each product using sterile forceps and scalpels and stored in 1.5 ml microcentrifuge tubes at −70 °C. These subsamples were shipped to the Biodiversity Institute of Ontario at the University of Guelph for DNA extraction and sequencing.

### DNA extraction

For each authenticated or commercial sample, one gram of tissue/product was divided into 10 MP lysing matrix tubes “A” (100 mg each) and homogenized using an MP FastPrep-24 Instrument (MP Biomedicals Inc.) at speed 6 for 40 S. Total DNA of this homogenized slurry was extracted using the Nucleospin tissue kit (Macherey-Nagel Inc.) following the manufacturer’s instructions and eluted in 50 μl of molecular biology grade water.

### DNA barcode library construction

The *COI* standard barcoding region (652 bp) was amplified for each of the 96 authenticated samples using a pair of newly designed degenerate fish primers (Table 1) as well as a primer cocktail previously described^27^. Each amplification reaction contained 2 μl DNA template, 17.5 μl molecular biology grade water, 2.5 μl 10X reaction buffer, 1 μl MgCl2 (50 μM), 0.5 μl dNTPs mix (10 mM), 0.5 μl forward primer (10 μM), 0.5 μl reverse primer (10 μM), and 0.5 μl Invitrogen’s Platinum Taq polymerase (5 U/μl) in a total volume of 25 μl. The PCR conditions were initiated with a heated lid at 95 °C for 5 min, followed by a total of 35 cycles of 94 °C for 40 S, 51 °C for 1 min, and 72 °C for 30 S, and a final extension at 72 °C for 5 min, and hold at 4 °C. PCR reactions were carried out using Mastercycler ep gradient S (Eppendorf, Mississauga, ON, Canada) thermal cyclers. PCR success was verified by 1.5% agarose gel electrophoresis. A DNA template negative control reaction was included in all experiments to test for contamination. Two microliters of each amplicon were subsequently used directly for bi-directional Sanger sequencing with the M13 primers described in Table 1 using Applied Biosystems’s BigDye Terminator chemistry V3.1 (Foster City, CA, USA). Sequencing reactions were cleaned using EdgeBio’s AutoDTR96 (Gaithersburg, MD, USA) and visualized on an ABI 3730xl sequencer (Applied Biosystems)^28^,^29^. Sequence editing and contig assembly were carried out using CodonCode Aligner v 3.7.1.1 (CodonCode Corp., Dedham, MA, USA). Identification of the tested samples was conducted using BLAST in GenBank and a local barcode library for selected taxa with a minimum BLAST cut off of 98% identity for a top match. The accession numbers of the generated sequences are available in the Supplementary Materials-Table S1.

### PCR primer design and *in silico* testing

A total of 8845 fish *COI* barcodes were downloaded from GenBank (n = 1894) and the Barcode of Life Database (BOLD; n = 6951) using the FDA Seafood List (http://www.accessdata.fda.gov/scripts/fdcc/?set=seafoodlist) as a guide for the target species. All sequences were aligned and multiple copies of identical sequences were removed. Degenerate nucleotides and inosine were used to manually design a fish *COI* primer set to amplify 652 bp—the standard barcoding region—within a wide range of fish species (Table 1).

**Figure 2:**
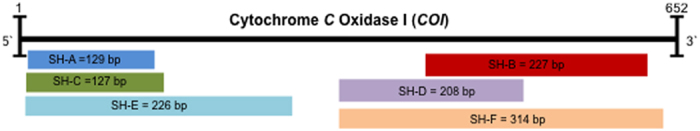
Schematic representation of regions amplified by the mini-barcode primers designed in this study, shown within the standard *COI* barcode region .

The newly designed *COI* primer set was used to amplify the full-length DNA barcode in the 96 authenticated samples from the FDA. For comparison, a previously designed primer cocktail was also used to amplify these samples^27^. The *COI* sequences generated from the authenticated samples, along with the unique *COI* sequences downloaded from GenBank and BOLD, were then used to design multiple mini-barcode primer sets to amplify partial fragments within the standard *COI* barcoding region (Fig. 2). The primers were picked according to the availability of highly-conserved priming sites in a wide range of species with consideration of the primer stability in PCR reactions as well as the physical and structural properties of oligos (e.g., annealing temperature, G+C percentage, hairpin formation, and self- and hetero-dimer formation). *In silico* analysis was also carried out using UCLUST^30^ and MEGA V5.2.2^31^ on the newly designed mini-barcode primers to assess the potential for the amplification targets to differentiate fish species at the 98% and 100% levels (Table 2 and Table S2). The analysis included full-length DNA barcodes representing 200 species and 124 genera obtained from the FDA’s Reference Standard Sequence Library for Seafood Identification. M13 forward and reverse tails were attached to the forward and reverse barcoding primers, respectively, to facilitate high-throughput sequencing. The Integrated DNA Technologies (IDT) analysis tool was used to evaluate all the mentioned parameters^32^. Six mini-barcode primer sets were selected (Table 1) for further testing with commercial samples.

### Mini-barcoding PCR Optimization Strategy

The amplification conditions for all the primer sets were tested using a gradient PCR approach at a wide range of annealing temperatures (43–60 °C). The composition of the amplification reactions, the PCR amplification conditions (except the annealing temperature), and the sequencing conditions were exactly the same as those used previously for amplification and sequencing of the full-length barcode. The optimal annealing temperature of each primer set was determined based on the results of gel electrophoresis of temperature gradient PCR products and is listed in Table 1. The mini-barcode amplification and sequencing steps were carried out on DNA from the 44 commercial fish products using each of the designed six sets of mini-barcode primers. Reagent blanks and a non-template PCR control were included in all PCR and sequencing experiments. Sequence editing and contig assembly of the generated barcodes were carried out as described for the full-length barcodes using CodonCode Aligner v 3.7.1.1 (CodonCode Corp., Dedham, MA, USA). Species identification for each sample was conducted using BLAST against GenBank and a local barcode library for selected taxa with a minimum BLAST cut off of 98% identity for a top match. These results were verified by neighbour-joining analysis^33^ and subsequent evaluation of the grouping of specimens tested as compared to database sequences^5^.

## Results and Discussion

Full-length DNA barcodes (652 bp) could be recovered using the newly-designed Fish primers (Fish UnivF and Fish UnivR) in 86 out of 88 of fish species (93 out of 96 specimens) within the authenticated fish muscle tissue sample collection obtained from the FDA. Both peak intensities and sequencing qualities of the generated barcodes were compared to the sequences generated with the previously used primer cocktail^27^. The new full-length barcode fish primer set showed slightly higher success rate (97.7%) among the wide variety of the tested species compared to a success rate of 95.5% for those species sequenced with the previously developed primer cocktail.

Regarding the commercial fish products, the tested products included a wide range of processed products packed as cans, tins, retort pouches, jars, or tubes (Fig. 1). These samples were all shelf-stable, preserved products that had experienced different levels of processing, for instance, smoking, salting, etc., and they also contained multiple additives, preservatives, and flavors (Table 3). These traits may negatively impact the quantity and quality of DNA extracted from these samples, which can decrease subsequent DNA barcode recovery.

The standard *COI* barcoding of the 44 tested fish processed products was achieved in only 9 products (20.5%) using both the newly designed universal fish primer set (Table 1) and the previously used fish primer cocktail^27^. These full-length barcodes were generated from a variety of samples with different levels of processing and with a variety of additives (Table 3). The major cause of full-barcode failure was most likely due to the degradation of the DNA extracted from these samples as a result of different levels of processing and the presence of multiple additives^25^. Samples showed low amplicon yields in gel electrophoresis and poor quality sequences with co-amplification of multiple non-targets (results are not shown).

Previous work has shown the applicability of a mini-barcoding approach in different groups of organisms^21^,^34^,^35^. Furthermore, it has been shown that the sequencing information of any 100 bases or more within the standard *COI* barcoding region can distinguish up to 91–94% of species in different taxonomic groups^24^,^25^,^36^. Here, we developed primers to amplify 6 mini-barcodes for commercial fish species, based on species described in the FDA Seafood List. The target fragment size ranges between 127 bp and 314 bp (Fig. 2). When compared *in silico* using DNA barcodes from authenticated FDA fish specimens representing 124 genera and 200 species, the mini-barcode amplification targets showed high levels of differentiation at both the species and genus levels (Table 4 and Table S2). Overall, primer sets SH-B, SH-D, SH-E, and SH-F showed the greatest ability to resolve sequences at the genus and species levels. All four of these primer sets showed high potential for use in fish species identification, with resolution at the species level for 98–100% of the species analyzed at the 100% sequence identity level and 98–99% of the species analyzed at the 99% sequence identity level. Figure 2 demonstrates the amplification regions of the designed mini-barcode primers within the full-length *COI* DNA barcode. These mini-barcodes target 5**′** (SH-A, SH-C, SH-E) and 3**′** (SH-B, SH-F) regions of the standard DNA barcode as well as the middle region (SH-E, SH-D, SH-F). Hence, their combination can maximize recovery of sequence information from across the full-length DNA barcode and should provide sufficient sequence information for species identification^24^. In support of this, when the results of the *in silico* taxonomic analyses for all six mini-barcode primer sets were combined, species-level resolution was possible in 100% of sequences analyzed (Table S2). However, it is important to note that this analysis was restricted to sequences from authenticated specimens representing 200 species of commercial fish. Incorporation of sequences from a wider number of fish species may lead to less definitive results and may require slight primer modifications.

Out of the 44 processed fish products tested with the mini-barcode primer sets, 41 products (93.2%) could be mini-barcoded with at least one primer set. Three samples (RB-1_94, RB-1_104, and RB-2_114) were negative in both standard barcoding and mini-barcoding with all primer sets (Table 3). These samples were all labelled as tuna products (2 retort pouches of light tuna and 1 can of albacore) and contained a variety of additives. Although they showed some amplification success with the mini-barcoding primers, all generated amplicons failed at the sequencing stage. Two of these samples were marinated with either lemon or sweet and spicy marinades, which may either interfere with PCR amplification or result in low amplicon yield which cannot be successfully sequenced. Alternatively, DNA barcode failure in these products may be due to the presence of more than one species, which can co-amplify and produce a mixed electrophorogram^37^,^38^. For the samples which have multiple closely related species, they generated overlapping traces at few specific sites in the electrophorogram which were called as ambiguous bases. Indeed, the light tuna products may very well have contained multiple species, as FDA regulations allow for multiple species to be used in the production of canned light tuna as long as the color of the tuna meat is not darker than Munsell value 5.3^39^ (FDA, 2013a).

As for the remaining samples (41 products), the 6 mini-barcode primer sets showed different success rates with each tested product group (Table 4). Overall, primer sets SH-B, SH-C, SH-D, and SH-E had significantly higher proportions of sequencing success compared to the full barcode (Z-test, two-sided, P values all <0.05). Primer set SH-E (226 bp) showed the highest success rate with 39 samples barcoded (88.6%). The two additional samples that failed with this primer set (RB-1_100, smoked sprat in oil and RB-1_101, chunk light tuna in water) were amplified and sequenced with other sets. Sample RB-1_101 was successfully sequenced only with primer set SH-C, which amplifies 130 bp, indicating the high degradation level in the DNA extracted from this sample. On the other hand, sample RB-1_100 was a can of smoked sprat that was successfully sequenced using primer sets SH-B, SH-D, and SH-F (208–314 bp). These results indicate either a lack of specificity at the SH-A/SH-C/SH-E primer binding sites for this sample or degradation of DNA towards the 5**′** end of the standard barcoding region, as only the mini-barcodes closer to the 3**′** end were amplified (Fig. 2). After SH-E, the primer set with the next-highest success rate was SH-D (208 bp), which achieved 63.6% sequencing success among the 41 samples, followed by primer set SH-B (227 bp) with 54.5% sequencing success. Among individual product groups, the primer sets showed a range of amplification and sequencing success rates (Table 4). For example, for the 13 tuna samples tested, primer set SH-E showed the greatest sequencing success (n = 9) followed by primer set SH-C (n = 8), while the remaining primer sets were only able to obtain sequences for 0–1 of the tuna samples. Interestingly, primer set SH-F showed the greatest PCR amplification success with the tuna samples (n = 10), but none of these amplicons were successfully sequenced. This is most likely due to co-amplification of non-target DNA along with the target DNA. On the other hand, within the eight sardine samples, the highest performing primer set was again SH-E, with amplification and sequencing success for all samples. Primer sets SH-F and SH-D were successful in amplification and sequencing of 7 out of the 8 sardine samples, while SH-C was only able to amplify and sequence one of the products (RB-3_132). Interestingly, this product was only successfully sequenced by one other primer set (SH-E). The only species group where SH-E showed a reduced sequencing success rate compared to the other primer sets was for sprat products, in which case primer sets SH-F, SH-D, and SH-B showed the highest success rate (3 out of 3 products), whereas SH-E and SH-C showed success with 2 out of 3 products. Based on the set of commercial products tested here, these results show primer set SH-E to be the most favorable for use in mini-barcoding. However, in instances where this primer set fails to amplify a target sequence, primer sets SH-D or SH-C may be reasonable alternatives (Table 4).

All barcoded products could be identified as the species listed on the product label except in three cases involving tuna products (see below) and in two cases where species substitution was detected (Table 3). Species substitution is a form of seafood fraud in which seafood is mislabelled on imported or exported products. In one case of species substitution detected with the mini-barcodes, a sample labelled as “Wild Alaskan salmon” (RB-1_102) was found to be mislabelled. This sample was expected to be a species of Pacific salmon (genus *Oncorhynchus*), but it was identified by mini-barcoding as Atlantic salmon (*Salmo salar*). Atlantic salmon is not commercially harvested in North America, but rather it is a farm-raised fish. Furthermore, farming of Atlantic salmon is not permitted in the state of Alaska (http://www.legis.state.ak.us/basis/statutes.asp?title=16#16.40.100). While some species of Pacific salmon are actually sold at a lower price than Atlantic salmon, wild-caught salmon has certain marketing advantages over farm-raised salmon^37^, which may be a driving incentive for this form of substitution. Indeed, substitution of farm-raised salmon for wild-caught salmon is one of the examples of known species substitution given by the FDA^40^ (FDA, 2013b). On the other hand, another sample labelled as “Wild Alaskan sockeye salmon” (RB-1_92) was found to correctly contain the Pacific salmon species stated on the label - *Oncorhynchus nerka*. In another instance of mislabelling, sample RB-3_131 was labelled as “Mackerel in tomato sauce”, but DNA mini-barcoding identified this sample as *Decapterus russelli*. While mackerel is an acceptable market name for a number of species, including *Scomber scombrus, Gasterochisma melampus*, and *Grammatorcynus bicarinatus*, it is not an acceptable market name for *D. russelli* according to the FDA Seafood List^41^ (FDA, 2013c). One of the vernacular names associated with this scientific name is mackerel scad, but the only acceptable market name for *D. russelli* in the U.S. is scad or Indian scad. Consistent with these findings, a previous study reported that *Decapterus* spp. are known to be substituted for other species of higher value in processed foods and that they share organoleptic properties with species of the genus *Scomber*, making them difficult to differentiate without laboratory analyses^42^.

As discussed above, the set of mini-barcode primers developed here were able to identify tuna at the genus or species level for 10 out of the 13 processed tuna products. The three un-sequenced tuna samples could be amplified with at least one *COI* mini-barcode primer set, but failed in the Sanger sequencing step possibly due to the presence of multiple species in each sample, co-amplification of non-target, or due to DNA degradation. All the tested tuna samples were identified as belonging to the genus *Thunnus* except sample RB-3_122 which was identified as *Katsuwonus pelamis* (skipjack tuna). However, it is important to note that it was challenging to discriminate between closely related tuna species and these three products showed multiple *Thunnus* species matches at the ≥99% level. As a result, the species identifications did not match what was stated on the label for three of the products (RB-1_97, RB-3_125, and RB-3_127). Although the *in silico* analysis showed high levels of species resolution (Table 2 and Table S2), the group of sequences tested only included two *Thunnus* species (*T. alalunga* and *T. albacares*). Based on these results, COI mini-barcoding may be used for the identification of tuna at the genus level but it is not recommended for the reliable differentiation of species within the *Thunnus* genus. Previous studies have also reported difficulties in differentiating closely related *Thunnus* species using DNA barcoding of the *COI* marker only^43^,^44^. To overcome this challenge, we recommend using additional genetic markers for further authentication of tuna samples at the species level^23^,^38^.

Besides detecting instances of species mislabelling, DNA mini-barcoding can also be used to clarify the identity of species in products with nonspecific labels. For example, a sample labelled as “Smoked garlic pepper salmon” (RB-2_106), with no species names listed, was found to contain Atlantic salmon by DNA mini-barcoding. Additionally, for the two samples of fish balls (RB-1_91 and RB-3_129), the ingredients list on the label simply stated “fish meat” (61%) and claimed the presence of one or more of the following: *Gadus morhua*, *Melanogrammus aeglefinus*, *Pollachius virens*, or *Merluccius merluccius*. These two samples were successfully mini-barcoded and identified with at least 3 primer sets as *Melanogrammus aeglefinus* (Haddock) only.

Although regulations for the safety and quality of commercial seafood exist in North America, the enforcement of proper species labelling has proven to be particularly difficult in heavily processed seafood products. This study sets the stage for the use of DNA information to identify a wide range of fish species in heavily processed products using one or more mini-barcode primer pairs. Basing the mini-barcode primer design on sequences of the full-length DNA barcode has allowed us to build upon the extensive research that has been carried out in this field^9^,^12^, including a database that contains DNA barcodes for over 10,000 fish species (i.e., www.fishbol.org).

## Conclusion

This study presents a DNA mini-barcoding system for species identification applicable to heavily processed fish products. Six mini-barcode primer sets were developed, with one primer set in particular showing a high rate of success for identification of heavily processed products at the species or genus level. The additional primer sets developed showed promise as supplemental tools to be used in cases where the initial primer set fails. All mini-barcode primer sets showed increased performance for species identification in heavily processed products as compared to full-length DNA barcode primers. Additionally, the mini-barcoding system provides a new avenue for the utility of next-generation DNA sequencing for authentication of mixed products that may contain multiple species and experienced different levels of DNA un-friendly commercial procedures. Overall, the mini-barcode system developed here provides a means to identify species in heavily processed products and may be used for the detection and enforcement of species substitution on the commercial market.

## Additional Information

**How to cite this article**: Shokralla, S. *et al.* A DNA Mini-Barcoding System for Authentication of Processed Fish Products. *Sci. Rep.* **5**, 15894; doi: 10.1038/srep15894 (2015).

## Supplementary Material

Supplementary Information

## Figures and Tables

**Table 1:** PCR amplification and sequencing primers used for DNA mini-barcoding of the processed fish products.

Primer Set	Primer name	Direction	Primer sequence (5′-3′)	Barcode length (bp)	Annealing temp. (°C)
Universal Fish	Fish_Univ_F	Forward	CACGACGTTGTAAAACGACACYAAICAYAAAGAYATIGGCAC	652	51
	Fish_Univ_R	Reverse	GGATAACAATTTCACACAGGACITCAGGGTGWCCGAARAAYCARAA		
Mini_SH-A	Fish_miniA_F_t	Forward	CACGACGTTGTAAAACGACACIAAICAIAAAGAYATYGGC	129	46
	Fish_miniA_R_t	Reverse	GGATAACAATTTCACACAGGAARAAAATYATAACRAAIGCRTGIGC		
Mini_SH-B	Fish_miniB_F_t	Forward	CACGACGTTGTAAAACGACGCIGGIRTYTCITCIATYYTAG	227	48
	Fish_miniB_R_t	Reverse	GGATAACAATTTCACACAGGACTTCAGGGTGICCGAARAATCA		
Mini_SH-C	Fish_miniC_F_t	Forward#	CACGACGTTGTAAAACGACACYAAICAYAAAGAYATIGGCAC	127	46
	Fish_miniC_R_t	Reverse	GGATAACAATTTCACACAGGGAARATCATAATGAAGGCATGIGC		
Mini_SH-D	Fish_miniD_F_t	Forward*	CACGACGTTGTAAAACGACGGIACIGGITGRACIGTITAYCCYCC	208	50
	Fish_miniD_R_t	Reverse	GGATAACAATTTCACACAGGGTRATICCIGCIGCIAGIAC		
Mini_SH-E	Fish_miniE_F_t	Forward#	CACGACGTTGTAAAACGACACYAAICAYAAAGAYATIGGCAC	226	46
	Fish_miniE_R_t	Reverse	GGATAACAATTTCACACAGGCTTATRTTRTTTATICGIGGRAAIGC		
Mini_SH-F	Fish_miniF_F_t	Forward*	CACGACGTTGTAAAACGACGGIACIGGITGRACIGTITAYCCYCC	314	49
	Fish_miniF_R_t	Reverse	GGATAACAATTTCACACAGGCTTCAGGGTGICCGAARAATC		
M13	M13F (-21)	Forward	CACGACGTTGTAAAACGAC	NA	NA
	M13R (-27)	Reverse	GGATAACAATTTCACACAGG		

^#^The forward sequence for primer set C is the same as the forward sequence for primer set E

^*^The forward sequence for primer set F is the same as the forward sequence for primer set D & Barcode length is calculated without amplification primers

**Table 2:** *In silico* analyses of the taxonomic resolution achieved by the six mini-barcoding regions when compared across 200 species from 124 genera using DNA barcodes from authenticated FDA reference samples.

*CO1* gene fragment	Size (bp)	Resolution at 100% identity				≤2% Nucleotide difference			
		#of Genera	%	#of Species	%	#of Genera	%	#of Species	%
Full barcode	652	124	100	200	100	124	100	185	92.5
A-fragment	129	124	100	191	95.5	122	98.4	178	89
B-fragment	227	124	100	200	100	124	100	194	97
C-fragment	127	121	97.6	188	94	119	96	174	87
D-fragment	208	124	100	200	100	124	100	192	96
E-fragment	226	124	100	198	99	122	98.4	177	88.5
F-fragment	314	124	100	200	100	124	100	195	97.5

Resolution at the 98% and 100% sequence identity levels.

**Table 3:** Results of commercially processed fish products tested with the DNA mini-barcoding system developed in this study.

Sample ID	Sample information					>550 bp	DNA mini-barcoding results							
	Product description on label	Fish type	Packed in	Processing type	Source		SH-A	SH-B	SH-C	SH-D	SH-E	SH-F	BLAST identification*	Notes
RB-1_90	Kipper fillets in brine	Herring	Brine	Tin	Ireland			√		√	√		*Clupea harengus*	P
RB-1_91	Fishballs in bouillon	Fish balls	Bouillon	Can	Sweden				√	√	√		*Melanogrammus aeglefinus*	P
RB-1_92	Wild Alaskan sockeye salmon	Salmon, Sockeye	Salt	Can	USA	√		√	√	√	√	√	*Oncorhynchus nerka*	P
RB-1_93	Premium skinless, boneless pink salmon	Salmon, pink	Water, salt	Retort pouch	Thailand	√		√		√	√		*Oncorhynchus gorbuscha*	P
RB-1_94	Gourmet albacore in olive oil	Tuna, Albacore	Olive oil	Can	USA	No sequence								
RB-1_95	Tuna fillets in olive oil	Tuna, Yellowfin	Olive oil	Jarred	Costa Rica				√	√	√		*Thunnus albacares*	P
RB-1_96	Moroccan sardines	Sardines	Oil	Tin	Morocco		√			√	√	√	*Sardina pilchardus*	P
RB-1_97	Tuna fillets with garlic in olive oil	Tuna, Yellowfin	Olive oil	Jarred	Costa Rica				√		√		*Thunnus atlanticus*	F
RB-1_98	Anchovy fillets in olive oil with capers	Anchovy	Olive oil, capers	Glass jar	Italy	√	√	√		√	√	√	*Engraulis encrasicolus*	P
RB-1_99	Anchovy fillets in olive oil, salt added	Anchovy	Olive oil, salt	Tin	Morocco	√	√	√	√	√	√	√	*Engraulis encrasicolus*	P
RB-1_100	Smoked sprats in oil	Sprat	Veg. oil, Onion	Can	Latvia			√		√		√	*Sprattus sprattus*	P
RB-1_101	Chunk light tuna in water	Tuna, Light	Water	Can	Not given				√				*Thunnus sp*	P
RB-1_102	Wild Alaskan salmon	Salmon, unspecified	Oil, vegetables	Can	France			√	√	√	√		*Salmo salar*	F
RB-1_103	Sardines in tomato sauce	Sardines	Tomato Sauce	Tin	Spain	√	√	√		√	√	√	*Sardina pilchardus*	P
RB-1_104	Sweet spicy marinated chunk light tuna	Tuna, Light	Seasoning	Retort pouch	Ecuador	No sequence								
RB-1_105	Premium Coho Salmon	Salmon, Coho	Unknown	Can	Not given						√		*Oncorhynchus kisutch*	P
RB-2_106	Smoked garlic pepper salmon	Salmon, unspecified	Garlic, Pepper	Can	Not given					√	√		*Salmo salar*	P
RB-2_107	Sardines in vegetable Oil	Sardines	Soybean oil	Tin	Croatia	√		√		√	√	√	*Sardina pilchardus*	P
RB-2_108	Sardines in olive oil	Sardines	Olive Oil	Tin	Portugal	√	√	√		√	√	√	*Sardina pilchardus*	P
RB-2_109	Smoked sprats in oil	Sprat	Veg. oil, lemon	Can	Latvia			√	√	√	√	√	*Sprattus sprattus*	P
RB-2_110	Chunk white albacore tuna in water	Tuna, Albacore	Water	Can	Not given				√		√		*Thunnus alalunga*	P
RB-2_111	Mackerel salad picnic with oil-vegetables	Mackerel	Oil, vegetables	Tin	Slovenia		√	√		√	√	√	*Scomber scombrus*	P
RB-2_112	Sardines with lemon	Sardines	Oil, lemon	Tin	Croatia		√	√		√	√	√	*Sardina pilchardus*	P
RB-2_113	Chunk light tuna in water pouch	Tuna, Light	Water	Retort pouch	Ecuador						√		*Thunnus tonggol*	P
RB-2_114	Zesty lemon pepper - chunk light tuna	Tuna, Light	Water, seasoning	Retort pouch	Ecuador	No sequence								
RB-2_115	Sardine in olive oil with lemon	Sardines	Olive Oil, Lemon	Tin	Portugal	√	√	√		√	√	√	*Sardina pilchardus*	P
RB-2_116	White tuna in olive oil	Tuna, Albacore	Olive oil	Can	Spain				√	√	√		*Thunnus alalunga*	P
RB-2_117	Herring fillets in paprika sauce	Herring	Paprika sauce	Tin	Germany			√		√	√		*Clupea harengus*	P
RB-2_118	Premium Alaskan pink salmon	Salmon, Pink	Unknown	Can	USA			√		√	√		*Oncorhynchus gorbuscha*	P
RB-2_119	Herring fillets in mustard sauce a la dijon	Herring	Mustard Sauce	Tin	Germany			√	√	√	√		*Clupea harengus*	P
RB-3_120	Anchovy paste	Anchovy	Anchovy Paste	Tube	USA			√	√	√	√		*Sardina pilchardus*	P
RB-3_121	Smoked wine maple salmon	Salmon, unspecified	Wine-maple	Can	Not given					√	√		*Salmo salar*	P
RB-3_122	Chunk light tuna in vegetable oil	Tuna, Light	Veg. Oil	Can	Not given						√		*Katsuwonus pelamis*	P
RB-3_123	Sardines in olive oil with chili peppers	Sardines	Olive oil, Chili	Tin	Portugal		√	√		√	√	√	*Sardina pilchardus*	P
RB-3_124	Chunk white albacore tuna	Tuna, Albacore	Water	Can	Ecuador				√		√		*Thunnus alalunga*	P
RB-3_125	Yellowfin tuna fillets packed in olive oil	Tuna, Yellowfin	Olive oil	Tin	Spain		√	√	√		√		*Thunnus obesus*	F
RB-3_126	Smoked sprats in oil	Sprat	Veg. oil, Spices	Can	Latvia			√	√	√		√	*Sprattus sprattus*	P
RB-3_127	Solid white albacore tuna	Tuna, Albacore	Olive oil	Can	USA				√		√		*Thunnus thynnus*	F
RB-3_128	Premium Chinook Salmon	Salmon, Chinook	Unknown	Can	Not given	√		√		√	√	√	*Oncorhynchus tshawytscha*	P
RB-3_129	Fishballs in bouillon	Fishballs	Bouillon	Can	Sweden		√			√	√		*Melanogrammus aeglefinus*	P
RB-3_130	Mackerel fillets in olive oil	Mackerel	Olive oil	Tin	Portugal		√	√			√		*Scomber japonicus*	P
RB-3_131	Mackerel in tomato sauce	Mackerel	Tomato Sauce	Can	Thailand			√			√		*Decapterus russelli*	F
RB-3_132	Seasoning for macoroni with sardines	Sardines	Seasoning	Can	Italy				√		√		*Sardina pilchardus*	P
RB-3_133	Herring fillets in dill herbs crème	Herring	Dill Herbs Crème	Tin	Germany				√	√	√	√	*Clupea harengus*	P

(√) Barcode recovered (*) BLAST results report the top bit score hit

(P) Identified species matches the product label (F) Identified species does not match the product label.

**Table 4:** Evaluation of amplification and sequencing success rates of *COI* full and mini-barcoding primer sets among all the tested commercial fish products (n = 44).

Samples	No	Amplification %							Sequencing %						
		>550 bp	SH-A	SH-B	SH-C	SH-D	SH-E	SH-F	>550 bp	SH-A	SH-B*	SH-C*	SH-D*	SH-E*	SH-F
Anchovy	3	100	100	100	66.7	100	100	100	66.7	66.7	100	66.7	100	100	66.7
Fishballs	2	50	100	100	50	100	100	100	0	50	0	50	100	100	0
Herring	4	0	0	100	50	100	100	100	0	0	75	50	100	100	25
Mackerel	3	0	100	100	0	66.7	100	66.7	0	66.7	100	0	33.3	100	33.3
Salmon	8	37.5	62.5	50	25	87.5	100	75	37.5	0	62.5	25	87.5	100	25
Sardines	8	50	87.5	87.5	12.5	87.5	100	87.5	50	75	75	12.5	87.5	100	87.5
Sprat	3	0	0	100	66.7	100	66.7	100	0	0	100	66.7	100	66.7	100
Tuna	13	23.1	7.7	7.7	61.5	46.2	69.2	76.9	0.0	7.7	7.7	61.5	7.7	69.2	0.0
Total	44	31.8	47.7	61.4	40.9	77.3	88.6	84.1	20.5	27.3	54.5	40.9	63.6	88.6	36.4

*indicates significantly higher proportions of sequencing success compared to the full barcode (Z-test, two-sided, P values all < 0.05).
