# Supplementary material for: A DNA Mini-Barcoding System for Authentication of Processed Fish Products
Source: Sci Rep. 2015 Oct 30;5:15894. doi: 10.1038/srep15894 (PMC4626862; doi:10.1038/srep15894)
Supplement: Supplementary Information [file srep15894-s1.pdf]

Supplementary Materials:

A DNA Mini-Barcoding System for Authentication of Processed Fish Products

Shadi Shokralla[1, 2,*], Rosalee S. Hellberg [3,*], Sara M. Handy[4], Ian King[1] and Mehrdad Hajibabaei[1]

[1]Biodiversity Institute of Ontario and Department of Integrative Biology, University of Guelph, Guelph, ON N1G 2W1, Canada.

[2]Department of Microbiology, Mansoura University, Mansoura 35516, Egypt.

[3]Chapman University, Schmid College of Science and Technology, Orange, CA 92866, USA.

[4]Office of Regulatory Science, Center for Food Safety and Applied Nutrition, U.S. Food and Drug Administration, College Park, MD 20740, USA.

*These authors contributed equally to this work. Correspondence and requests for materials should be addressed to S.S. (email: sshokral@uoguelph.ca).

Table S1 The U.S. Food and Drug Administration (FDA) authenticated fish tissue samples used for primers designing and testing

Sample ID	Species ID	Genbank Accession number	Sample ID	Species ID	Genbank Accession number	Sample ID	Species ID	Genbank Accession number	Sample ID	Species ID	Genbank Accession number
**FDA 14**	*Rhomboplites aurorubens*	HQ025004	**FDA 44**	*Echeneis neucratoides*	KF461171	**FDA 74**	*Lagocephalus laevigatus*	KF461191	**FDA 106**	*Carcharhinus isodon*	KF461150
**FDA 15**	*Morone saxatilis*	HQ024976	**FDA 45**	*Centropristis ocyurus*	KF461154	**FDA 75**	*Brama dussumieri*	KF461140	**FDA 107**	*Carcharhinus leucas*	KF461151
**FDA 17**	*Paralichthys lethostigma*	HQ024992	**FDA 46**	*Rypticus saponaceus*	KF461229	**FDA 76**	*Paralichthys lethostigma*	KF461217	**FDA 108**	*Dasyatis sabina*	KF461169
**FDA 18**	*Morone americana*	HQ024972	**FDA 47**	*Prionotus rubio*	KF461223	**FDA 77**	*Rhomboplites aurorubens*	KF461227	**FDA 109**	*Carcharhinus acronotus*	KF461148
**FDA 19**	*Centropristis striata*	HQ024936	**FDA 48**	*Chilomycterus schoepfii*	KF461157	**FDA 78**	*Balistes vetula*	KF461139	**FDA 134**	*Mugil cephalus*	n/a*
**FDA 20**	*Oreochromis niloticus*	HQ024988	**FDA 49**	*Lutjanus campechanus*	KF461194	**FDA 79**	*Chaetodipterus faber*	KF461156	**FDA 135**	*Epinephalus flavolimbatus*	n/a*
**FDA 21**	*Urophycis chuss*	HQ025024	**FDA 50**	*Lutjanus synagris*	KF461200	**FDA 80**	*Scomberomorus cavalla*	KF461231	**FDA 136**	*Mycteroperca bonaci*	n/a*
**FDA 22**	*Merluccius bilinearis*	HQ024964	**FDA 52**	*Hemanthias leptus*	KF461187	**FDA 81**	*Pareques umbrosus*	KF461219	**FDA 146**	*Symphorichthys spilurus*	n/a*
**FDA 23**	*Micropogonias undulatus*	HQ024968	**FDA 54**	*Helicolenus dactylopterus*	KF461186	**FDA 82**	*Caulolatilus chrysops*	KF461153	**FDA 147**	*Macolor macularis*	n/a*
**FDA 25**	*Leiostomus xanthurus*	HQ024956	**FDA 55**	*Kyphosus incisor*	KF461190	**FDA 83**	*Trachinotus carolinus*	KF461244	**FDA 151**	*Diagramma pictum*	n/a*
**FDA 26**	*Stenotmus chrysops*	HQ025020	**FDA 56**	*Bagre marinus*	KF461135	**FDA 84**	*Selene setapinnis*	KF461235	**FDA 153**	*Lutjanus rivulatus*	n/a*
**FDA 27**	*Pomatomus saltatrix*	HQ025000	**FDA 57**	*Elops saurus*	KF461177	**FDA 86**	*Euthynnus alletteratus*	KF461180	**FDA 156**	*Epinephelus polyphekadion*	n/a*
**FDA 24**	*Salmo salar*	HQ025008	**FDA 58**	*Lutjanus griseus*	KF461196	**FDA 87**	*Lobotes surinamensis*	KF461193	**FDA 158**	*Lutjanus argentimaculatus*	n/a*
**FDA 29**	*Ocyurus chrysurus*	HQ024984	**FDA 59**	*Balistes capriscus*	KF461137	**FDA 88**	*Scomberomorus maculatus*	KF461233	**FDA 159**	*Epinephelus undulosus*	n/a*
**FDA 32**	*Scomberomorus regalis*	HQ025016	**FDA 60**	*Hyperoglyphe bythites*	KF461189	**FDA 89**	*Ophichthus gomesii*	KF461209	**FDA 160**	*Lutjanus bohar*	n/a*
**FDA 33**	*Mugil cephalus*	HQ024980	**FDA 61**	*Calamus leucosteus*	KF461142	**FDA 90**	*Centropristis ocyurus*	KF461155	**FDA 162**	*Epinephelus coioides*	n/a*
**FDA 34**	*Peprilus paru*	HQ024996	**FDA 62**	*Pomatomus saltatrix*	KF461220	**FDA 91**	*Pagrus pagrus*	KF461214	**FDA 164**	*Lutjanus malabaricus*	n/a*
**FDA 35**	*Haemulon plumierii*	HQ024940	**FDA 63**	*Archosargus probatocephalus*	KF461133	**FDA 92**	*Scorpaena dispar*	KF461234	**FDA 165**	*Lutjanus synagris*	n/a*
**FDA 36**	*Lagodon rhomboides*	HQ024952	**FDA 64**	*Selene vomer*	KF461236	**FDA 95**	*Cynoscion arenarius*	KF461163	**FDA 166**	*Epinephelus guttatus*	n/a*
**FDA 37**	*Archosargus probatocephalus*	HQ024932	**FDA 66**	*Rachycentron canadum*	KF461225	**FDA 97**	*Haemulon aurolineatum*	KF461185	**FDA 168**	*Cynoscion nebulosus*	KF461166
**FDA 38**	*Sciaenops ocellatus*	HQ025012	**FDA 70**	*Lutjanus griseus*	KF461197	**FDA 98**	*Chloroscombrus chrysurus*	KF461158	**FDA 169**	*Corniger spinosus*	KF461160
**FDA 39**	*Ictalurus punctatus*	HQ024944	**FDA 71**	*Canthidermis maculata*	KF461143	**FDA 99**	*Ariopsis felis*	KF461134	**FDA 170**	*Synodus foetens*	KF461243
**FDA 42**	*Elagatis bipinnulata*	KF461174	**FDA 72**	*Pristigenys alta*	KF461224	**FDA 100**	*Sphyrna tiburo*	KF461242	**FDA 178**	*Lepidocybium flavobrunneum*	KF461192
**FDA 43**	*Opsanus pardus*	KF461213	**FDA 73**	*Holocentrus rufus*	KF461188	**FDA 102**	*Carcharhinus brevipinna*	KF461149	**FDA 182**	*Conger oceanicus*	KF461159

*Sequences located here: http://www.accessdata.fda.gov/scripts/fdcc/?set=seafood_barcode_data

Table S2 Detailed results of *in silico* analysis of the taxonomic resolution achieved by the six mini-barcoding regions when compared across 200 species from 124 genera using DNA barcodes from authenticated FDA reference samples. Analysis was carried out based on sequence identity cut-off levels of 98% and 100%

98%	FDA	Primer sets							FDA	Primer sets							FDA	Primer sets					
	Species	A	B	C	D	E	F		Species	A	B	C	D	E	F		Species	A	B	C	D	E	F
1	Aethaloperca rogaa							41	Diagramma pictum							81	Lethrinus erythraeanthus						
2	Alectis ciliaris							42	Dissostichus eleginoides							82	Lethrinus lentjan						
3	Anarhichas lupus							43	Echeneis neucratoides							83	Lethrinus microdon						
4	Aprion virescens							44	Echiophis punctifer							84	Limanda ferruginea						
5	Archosargus probatocephalus							45	Elagatis bipinnulata							85	Lobotes surinamensis						
6	Ariopsis felis							46	Elops saurus							86	Lophius americanus						
7	Bagre marinus							47	Eopsetta jordani							87	Lophius litulon						
8	Balistes capriscus							48	Epinephalus bleekeri							88	Lutjanus argentimaculatus						
9	Balistes vetula							49	Epinephalus flavolimbatus							89	Lutjanus bohar						
10	Brama dussumieri							50	Epinephalus maculatus							90	Lutjanus campechanus						
11	Brosme brosme							51	Epinephelus adscensionis							91	Lutjanus griseus						
12	Brotula barbata							52	Epinephelus coioides							92	Lutjanus guttatus						
13	Calamus leucosteus							53	Epinephelus drummondhayi							93	Lutjanus jocu						
14	Canthidermis maculata							54	Epinephelus guttatus							94	Lutjanus malabaricus						
15	Canthidermis sufflamen							55	Epinephelus polyphekadion							95	Lutjanus peru						
16	Caranx caninus							56	Epinephelus quoyanus							96	Lutjanus rivulatus						
17	Caranx crysos							57	Epinephelus rivulatus							97	Lutjanus synagris						
18	Caranx hippos							58	Epinephelus undulosus							98	Macolor macularis						
19	Carcharhinus acronotus							59	Euthynnus affinis							99	Malacanthus plumieri						
20	Carcharhinus brevipinna							60	Euthynnus alletteratus							100	Melanogrammus aeglefinus						
21	Carcharhinus isodon							61	Gadus macrocephalus							101	Menticirrhus americanus						
22	Carcharhinus leucas							62	Gadus morhua							102	Merluccius bilnearis						
23	Carcharhinus limbatus							63	Gempylus serpens							103	Micropogonias undulatus						
24	Caulolatilus chrysops							64	Glyptocephalus cynoglossus							104	Microstomus pacificus						
25	Centropristis ocyurus							65	Gymnothorax kolpos							105	Morone americana						
26	Centropristis striata							66	Haemulon aurolineatum							106	Morone saxatilis						
27	Cephalopholis argus							67	Haemulon plumieri							107	Mugil cephalus						
28	Cephalopholis fulva							68	Helicolenus dactylopterus							108	Muraena retifera						
29	Cephalopholis sonnerati							69	Hemanthias leptus							109	Mycteroperca bonaci						
30	Chaetodipterus faber							70	Holocentrus rufus							110	Mycteroperca microlepis						
31	Chanoschanos							71	Hyperoglyphe bythites							111	Narcine bancroftii						
32	Chilomycterus schoepfi							72	Ictalurus furcatus							112	Neomerinthe hemingwayi						
33	Chloroscombrus chrysurus							73	Ictalurus punctatus							113	Ocyurus chrysurus						
34	Conger oceanicus							74	Ictiobus cyprinellus							114	Oncorhynchus gorbuscha						
35	Corniger spinosus							75	Katsuwonus pelamis							115	Oncorhynchus kisutch						
36	Cynoscion arenarius							76	Kyphosus incisor							116	Oncorhynchus mykiss						
37	Cynoscion nebulosus							77	Lagocephalus laevigatus							117	Ophichthus gomesii						
38	Dasyatis americana							78	Lagodon rhomboides							118	Ophichthus rex						
39	Dasyatis sabina							79	Leiostomus xanthurus							119	Ophiodon elongatus						
40	Diagramma melanacrum							80	Lepidocybium flavobrunneum							120	Opsanus beta						

Recovered to species level

Recovered to genus level

Not recovered

	FDA	Primer sets							FDA	Primer sets					
98%	Species	A	B	C	D	E	F		Species	A	B	C	D	E	F
121	Opsanus pardus							161	Sebastes maliger						
122	Oreochromis mossambicus							162	Sebastes norvegicus						
123	Oreochromis niloticus							163	Sebastes pinniger						
124	Pagrus pagrus							164	Selene peruviana						
125	Pampus argenteus							165	Selene setapinnis						
126	Pangasius bocourti							166	Selene vomer						
127	Pangasius conchophilus							167	Semicossyphus pulcher						
128	Pangasius hypophthalmus							168	Seriola dumerili						
129	Paralichthys albigutta							169	Seriola lalandi						
130	Paralichthys lethostigma							170	Seriola rivoliana						
131	Paranthias furcifer							171	Siganus javus						
132	Pareques umbrosus							172	Sphoeroides nephelus						
133	Peprilus paru							173	Sphoeroides spengleri						
134	Platichthys stellatus							174	Sphoeroides testudineus						
135	Pleuronectes americanus							175	Sphyraena argentea						
136	Pleuronectes vetulus							176	Sphyraena barracuda						
137	Pogonias cromis							177	Sphyraena guachancho						
138	Pollachius virens							178	Sphyrna lewini						
139	Pomadasys kaakan							179	Sphyrna tiburo						
140	Pomatomus saltatrix							180	Stenotomus chrysops						
141	Prionotus rubio							181	Symphorichthys spilurus						
142	Pristigenys alta							182	Symphorus nematophorus						
143	Rachycentron canadum							183	Synodus foetens						
144	Reinhardtius stomias							184	Takifugu chinensis						
145	Rhizoprionodon terraenovae							185	Takifugu niphobles						
146	Rhomboplites aurorubens							186	Takifugu ocellatus						
147	Ruvettus pretiosus							187	Takifugu pardalis						
148	Rypticus saponaceus							188	Takifugu poecilonotus						
149	Salmo salar							189	Takifugu porphyreus						
150	Sciaenops ocellatus							190	Takifugu snyderi						
151	Scomber japonicus							191	Takifugu vermicularis						
152	Scomberomorus cavalla							192	Takifugu zanthopterus						
153	Scomberomorus maculatus							193	Theragra chalcogramma						
154	Scomberomorus regalis							194	Thunnus alalunga						
155	Scophthalmus aquosus							195	Thunnus albacares						
156	Scorpaena dispar							196	Trachinotus carolinus						
157	Sebastes aleutianus							197	Trichiurus lepturus						
158	Sebastes alutus							198	Urophycis chuss						
159	Sebastes entomelas							199	Variola louti						
160	Sebastes flavidus							200	Zeus faber						

Recovered to species level

Recovered to genus level

Not recovered

100%	FDA	Primer sets					
	Species	A	B	C	D	E	F
1	Aethaloperca rogaa						
2	Alectis ciliaris						
3	Anarhichas lupus						
4	Aprion virescens						
5	Archosargus probatocephalus						
6	Ariopsis felis						
7	Bagre marinus						
8	Balistes capriscus						
9	Balistes vetula						
10	Brama dussumieri						
11	Brosme brosme						
12	Brotula barbata						
13	Calamus leucosteus						
14	Canthidermis maculata						
15	Canthidermis sufflamen						
16	Caranx caninus						
17	Caranx crysos						
18	Caranx hippos						
19	Carcharhinus acronotus						
20	Carcharhinus brevipinna						
21	Carcharhinus isodon						
22	Carcharhinus leucas						
23	Carcharhinus limbatus						
24	Caulolatilus chrysops						
25	Centropristis ocyurus						
26	Centropristis striata						
27	Cephalopholis argus						
28	Cephalopholis fulva						
29	Cephalopholis sonnerati						
30	Chaetodipterus faber						
31	Chanoschanos						
32	Chilomycterus schoepfi						
33	Chloroscombrus chrysurus						
34	Conger oceanicus						
35	Corniger spinosus						
36	Cynoscion arenarius						
37	Cynoscion nebulosus						
38	Dasyatis americana						
39	Dasyatis sabina						
40	Diagramma melanacrum						
41	Diagramma pictum						
42	Dissostichus eleginoides						
43	Echeneis neucratoides						
44	Echiophis punctifer						
45	Elagatis bipinnulata						
46	Elops saurus						
47	Eopsetta jordani						
48	Epinephalus bleekeri						
49	Epinephalus flavolimbatus						
50	Epinephalus maculatus						
51	Epinephelus adscensionis						
52	Epinephelus coioides						
53	Epinephelus drummondhayi						
54	Epinephelus guttatus						
55	Epinephelus polyphekadion						
56	Epinephelus quoyanus						
57	Epinephelus rivulatus						
58	Epinephelus undulosus						
59	Euthynnus affinis						
60	Euthynnus alletteratus						
61	Gadus macrocephalus						
62	Gadus morhua						
63	Gempylus serpens						
64	Glyptocephalus cynoglossus						
65	Gymnothorax kolpos						
66	Haemulon aurolineatum						
67	Haemulon plumieri						
68	Helicolenus dactylopterus						
69	Hemanthias leptus						
70	Holocentrus rufus						
71	Hyperoglyphe bythites						
72	Ictalurus furcatus						
73	Ictalurus punctatus						
74	Ictiobus cyprinellus						
75	Katsuwonus pelamis						
76	Kyphosus incisor						
77	Lagocephalus laevigatus						
78	Lagodon rhomboides						
79	Leiostomus xanthurus						
80	Lepidocybium flavobrunneum						
81	Lethrinus erythraeanthus						
82	Lethrinus lentjan						
83	Lethrinus microdon						
84	Limanda ferruginea						
85	Lobotes surinamensis						
86	Lophius americanus						
87	Lophius litulon						
88	Lutjanus argentimaculatus						
89	Lutjanus bohar						
90	Lutjanus campechanus						
91	Lutjanus griseus						
92	Lutjanus guttatus						
93	Lutjanus jocu						
94	Lutjanus malabaricus						
95	Lutjanus peru						
96	Lutjanus rivulatus						
97	Lutjanus synagris						
98	Macolor macularis						
99	Malacanthus plumieri						
100	Melanogrammus aeglefinus						
101	Menticirrhus americanus						
102	Merluccius bilnearis						
103	Micropogonias undulatus						
104	Microstomus pacificus						
105	Morone americana						
106	Morone saxatilis						
107	Mugil cephalus						
108	Muraena retifera						
109	Mycteroperca bonaci						
110	Mycteroperca microlepis						
111	Narcine bancroftii						
112	Neomerinthe hemingwayi						
113	Ocyurus chrysurus						
114	Oncorhynchus gorbuscha						
115	Oncorhynchus kisutch						
116	Oncorhynchus mykiss						
117	Ophichthus gomesii						
118	Ophichthus rex						
119	Ophiodon elongatus						
120	Opsanus beta						

100%	FDA	Primer sets							FDA	Primer sets					
	Species	A	B	C	D	E	F		Species	A	B	C	D	E	F
121	Opsanus pardus							161	Sebastes maliger						
122	Oreochromis mossambicus							162	Sebastes norvegicus						
123	Oreochromis niloticus							163	Sebastes pinniger						
124	Pagrus pagrus							164	Selene peruviana						
125	Pampus argenteus							165	Selene setapinnis						
126	Pangasius bocourti							166	Selene vomer						
127	Pangasius conchophilus							167	Semicossyphus pulcher						
128	Pangasius hypophthalmus							168	Seriola dumerili						
129	Paralichthys albigutta							169	Seriola lalandi						
130	Paralichthys lethostigma							170	Seriola rivoliana						
131	Paranthias furcifer							171	Siganus javus						
132	Pareques umbrosus							172	Sphoeroides nephelus						
133	Peprilus paru							173	Sphoeroides spengleri						
134	Platichthys stellatus							174	Sphoeroides testudineus						
135	Pleuronectes americanus							175	Sphyraena argentea						
136	Pleuronectes vetulus							176	Sphyraena barracuda						
137	Pogonias cromis							177	Sphyraena guachancho						
138	Pollachius virens							178	Sphyrna lewini						
139	Pomadasys kaakan							179	Sphyrna tiburo						
140	Pomatomus saltatrix							180	Stenotomus chrysops						
141	Prionotus rubio							181	Symphorichthys spilurus						
142	Pristigenys alta							182	Symphorus nematophorus						
143	Rachycentron canadum							183	Synodus foetens						
144	Reinhardtius stomias							184	Takifugu chinensis						
145	Rhizoprionodon terraenovae							185	Takifugu niphobles						
146	Rhomboplites aurorubens							186	Takifugu ocellatus						
147	Ruvettus pretiosus							187	Takifugu pardalis						
148	Rypticus saponaceus							188	Takifugu poecilonotus						
149	Salmo salar							189	Takifugu porphyreus						
150	Sciaenops ocellatus							190	Takifugu snyderi						
151	Scomber japonicus							191	Takifugu vermicularis						
152	Scomberomorus cavalla							192	Takifugu zanthopterus						
153	Scomberomorus maculatus							193	Theragra chalcogramma						
154	Scomberomorus regalis							194	Thunnus alalunga						
155	Scophthalmus aquosus							195	Thunnus albacares						
156	Scorpaena dispar							196	Trachinotus carolinus						
157	Sebastes aleutianus							197	Trichiurus lepturus						
158	Sebastes alutus							198	Urophycis chuss						
159	Sebastes entomelas							199	Variola louti						
160	Sebastes flavidus							200	Zeus faber						

Recovered to species level

Recovered to genus level

Not recovered
